# Urinary Phthalate Metabolites and Biomarkers of Oxidative Stress in a Mexican-American Cohort: Variability in Early and Late Pregnancy

**DOI:** 10.3390/toxics4010007

**Published:** 2016-03-14

**Authors:** Nina Holland, Karen Huen, Vy Tran, Kelly Street, Brian Nguyen, Asa Bradman, Brenda Eskenazi

**Affiliations:** Center for Environmental Research and Children’s Health (CERCH), School of Public Health, University of California, Berkeley, 50 University Hall, Berkeley, CA 94720-7360, USA; khuen@berkeley.edu (K.H.); vytran91@gmail.com (V.T.); kstreet@berkeley.edu (K.S.); brianminh94@berkeley.edu (B.N.); abradman@berkeley.edu (A.B.); eskenazi@berkeley.edu (B.E.)

**Keywords:** phthalates, isoprostane, pregnancy, birth cohort, oxidative stress, endocrine disruptors, *in utero* exposure

## Abstract

People are exposed to phthalates through their wide use as plasticizers and in personal care products. Many phthalates are endocrine disruptors and have been associated with adverse health outcomes. However, knowledge gaps exist in understanding the molecular mechanisms associated with the effects of exposure in early and late pregnancy. In this study, we examined the relationship of eleven urinary phthalate metabolites with isoprostane, an established marker of oxidative stress, among pregnant Mexican-American women from an agricultural cohort. Isoprostane levels were on average 20% higher at 26 weeks than at 13 weeks of pregnancy. Urinary phthalate metabolite concentrations suggested relatively consistent phthalate exposures over pregnancy. The relationship between phthalate metabolite concentrations and isoprostane levels was significant for the sum of di-2-ethylhexyl phthalate and the sum of high molecular weight metabolites with the exception of monobenzyl phthalate, which was not associated with oxidative stress at either time point. In contrast, low molecular weight metabolite concentrations were not associated with isoprostane at 13 weeks, but this relationship became stronger later in pregnancy (*p*-value = 0.009 for the sum of low molecular weight metabolites). Our findings suggest that prenatal exposure to phthalates may influence oxidative stress, which is consistent with their relationship with obesity and other adverse health outcomes.

## 1. Introduction

Phthalates are a group of chemicals used in personal care products, including fragrances, cosmetics and shampoo. They are also utilized as plasticizers in flexible plastic products, such as food packaging, building materials and medical devices. Due to the non-covalent bonds with the plastics they soften, phthalates leach easily into the environment [1]. Human exposure occurs mainly via diet, inhalation and dermal absorption. Phthalates have short half-lives and are rapidly excreted in humans. Phthalate metabolites are almost universally present in human urine at varying levels in the U.S. population [1]. Monoethyl phthalate (MEP), a metabolite of diethyl phthalate (DEP), a fragrance solvent in personal care products, is generally detected at the highest concentrations compared to other metabolites [2].

Exposure to certain phthalates has been associated with adverse birth outcomes, inflammation and asthma [3,4,5,6,7]. The Chronic Hazard Advisory Panel (CHAP) of the U.S. Consumer Product Safety Commission has identified the developing fetus as the most vulnerable target of toxicity for phthalates [8]. A growing body of mechanistic, animal and human data suggests a strong link between phthalates and the biological pathways that influence obesity in adults [9,10,11] and children [6].

Oxidative stress occurs when the body’s antioxidant defenses are overwhelmed by the reactive oxygen species generated through metabolic processes. The reactions of such free radicals with lipids, proteins or nucleic acids can lead to tissue damage. Obesity is considered a condition of systemic oxidative stress [12,13,14]. Isoprostanes result from free radical-dependent peroxidation of fatty acids and are well-validated biomarkers of oxidative stress [15,16,17,18,19]. In adults, increased isoprostane levels have been associated with higher body mass index (BMI) [20,21], diabetes mellitus [22] and hypercholesterolemia [23]. Overweight children have higher levels of isoprostanes than normal weight children [17,18,24].

Growing evidence suggests phthalates can induce oxidative stress [25,26,27,28,29,30,31]. For instance, epidemiologic studies have reported links between urinary phthalates (e.g., mono-isobutyl phthalate (MiBP)) and phthalate metabolites (e.g., di-2-ethylhexyl phthalate (DEHP) and dibutyl phthalate (DBP)) with increased levels of lipid peroxidation, inflammation and decreased levels of antioxidants [32,33,34]. Prenatal phthalate exposure was also associated with increased oxidative stress in male rat offspring [35], providing some evidence of fetal programming by phthalate exposure. Limited data, however, are available on the effects of phthalates on isoprostane levels during pregnancy, a particularly sensitive period for phthalate exposure. In a recent study of pregnant women from Boston, concentrations of nine phthalate metabolites measured four times during pregnancy were significantly associated with isoprostane levels in urine [4]. Estimated differences were greater for monobenzyl phthalate (MBzP), mono-*n*-butyl phthalate (MBP) and MiBP in comparison with metabolites of DEHP. The same group of investigators also reported a statistically-significant relationship of nearly all phthalate metabolites with isoprostane in pregnant women in Puerto Rico [36]. However, most of the existing data are for urban cohorts, and less is known about phthalate exposure and oxidative stress in minority pregnant women from agricultural areas who may also have a low socioeconomic status and different lifestyle variables. Therefore, we have examined whether those results hold true in a rural, Mexican-American population.

The purpose of the present study is to determine the relationship of isoprostane levels in maternal urine in early and late pregnancy with *in utero* phthalate metabolite concentrations in participants of the Center for Health Assessment of Mothers and Children of Salinas (CHAMACOS), a longitudinal birth cohort study of Mexican-American farmworkers and their families.

## 2. Materials and Methods

### 2.1. Study Subjects

The CHAMACOS study has been conducted in the agricultural region of Salinas Valley, California, since 1999 [37]. At the time of enrollment, pregnant women (*N* = 601) were at least 18 years of age, at less than 20 weeks gestation, Spanish or English speaking and receiving prenatal care at the community clinics. Trained bilingual, bicultural staff members interviewed CHAMACOS women twice during pregnancy (~13 weeks and 26 weeks gestation) and obtained information on sociodemographic characteristics, reproductive and medical history, exposures during pregnancy, and lifestyle and environmental exposures.

Spot urine samples were collected from women at the time of both interviews. The timing during the day (between 8 a.m. and 7 p.m.) was recorded, since we were aware of its potential relationship with the concentrations of isoprostane and possibly phthalate metabolites. Samples were frozen at −80 °C until shipped on dry ice to the Centers for Disease Control and Prevention (CDC). Phthalate metabolite concentrations were measured at CDC in 432 maternal urinary samples at 13 weeks of gestation and for 417 mothers at 26 weeks. Four hundred women had samples at both time points. Isoprostane was quantified in a subset of women (*N* = 166 at 13 weeks and *N* = 180 at 26 weeks) randomly selected from those with phthalate measurements. Study protocols were approved by the University of California, Berkeley, and the CDC Committees for Protection of Human Subjects. Written informed consent was obtained from all mothers at the time of enrollment.

### 2.2. Isoprostane Analysis

A competitive enzyme-linked immunosorbent assay (ELISA) kit was used to determine levels of 8-isoprostane in urine (Oxford Biomedical Research, Rochester Hills, MI, USA) collected at 13 and 26 weeks gestation. Samples were randomized across plates and run in duplicate at the Children’s Environmental Health Laboratory, the University of California, Berkeley. Additional quality assurance/quality control (QA/QC) provisions included repeats of 5% of samples and blanks and internal lab controls with good reproducibility of isoprostane measurements (coefficient of variation <7%).

### 2.3. Phthalate Metabolite Measurements

Eleven phthalate metabolites were quantified in prenatal urine samples collected from mothers at ~13 and 26 weeks gestation as previously described [38]. They included three low molecular weight (LMW) metabolites (MEP, MBP, MiBP), four high molecular weight (HMW) metabolites of DEHP (mono(2-ethylhexyl) phthalate (MEHP), mono(2-ethyl-5-hydroxyhexyl) phthalate (MEHHP), mono(2-ethyl-5-oxohexyl) phthalate (MEOHP), mono(2-ethyl-5-carboxypentyl) phthalate (MECPP)) and four additional HMW metabolites of other parent phthalates (MBzP, mono(3-carboxypropyl) phthalate (MCPP), monocarboxyoctyl phthalate (MCOP), monocarboxynonyl phthalate (MCNP)). Measurements were performed using online solid phase extraction coupled with isotope dilution high-performance liquid chromatography-electrospray ionization-tandem mass spectrometry [39]. QC procedures included the use of laboratory blanks, calibration standards and spiked controls with high and low concentrations.

The limits of detection (LOD) for all metabolites were previously reported [39,40]. Concentrations below the LOD with no corresponding instrumental signal were imputed from a log-normal distribution using the “fill-in” method described in Lubin *et al.* [41]. For other concentrations below the LOD, the actual instrument reading value was used. Summary measurements (e.g., ∑LMW, ∑HMW and ∑DEHP) were created as described elsewhere [42]. Briefly, molar concentrations were calculated by dividing the concentration of each metabolite by its molecular weight. For each summary measure, the molar concentrations for each group were summed and then multiplied by the average molecular weight of the metabolites in that group to yield measurements expressed in µg/L.

Urinary dilution was accounted for by using either creatinine or specific gravity for each urine sample analyzed for isoprostane and phthalate metabolites. Here, we report results based on creatinine adjustment for better comparability with the National Health and Nutrition Examination Survey (NHANES) data [2] and because some of the women in our study were missing specific gravity data. However, creatinine levels have been associated with factors, such as muscle mass and age [43], and can change over the course of pregnancy [44], possibly influencing urinary adjustment of phthalate metabolite concentrations. Thus, we also report a sensitivity analysis accounting for specific gravity instead of creatinine.

Specific gravity was measured with a refractometer (National Instrument Company Inc., Baltimore, MD, USA), while urinary creatinine was determined using a commercially-available diagnostic enzyme method (Vitros CREA slides; Ortho Clinical Diagnostics, Raritan, NJ, USA). Creatinine-adjusted phthalate metabolite concentrations expressed in µg/g creatinine were calculated by dividing phthalate metabolite concentrations (µg/L) by creatinine levels (g/L). The following formula was utilized to produce specific gravity-adjusted concentrations (µg/L): *P*_c_ = *P*[(1.024 − 1)]/(*SG* − 1), where *P*_c_ is the specific gravity-adjusted concentration, *P* is the metabolite concentration (µg/L), 1.024 is the median specific gravity of all samples and *SG* is the specific gravity for the specific sample [45]. Creatinine adjusted phthalate metabolite concentrations were used for descriptive analyses and correlation calculations. However, in regression models, we used unadjusted phthalate metabolite concentrations and included maternal creatinine or specific gravity levels as a covariate in the model, because previous studies have shown that the use of creatinine or specific gravity-adjusted phthalate metabolite concentrations in regression models can introduce bias [46]. Further, it was shown that using creatinine for adjustment, as has been reported for the National Health and Nutrition Examination Survey (NHANES) [1], can also minimize the noise of the intra-individual variability of spot sample measurements [47,48].

### 2.4. Statistical Analyses

All urinary phthalate metabolite measurements were log_10_ transformed to approximate a normal distribution. We calculated Pearson’s correlation coefficient to examine the correlation of concentrations of phthalate metabolites and isoprostane between time points (13 *vs.* 26 weeks of pregnancy). We also examined mean differences between 13-week and 26-week phthalate metabolite concentrations using a paired *t*-test. We used analysis of variance (ANOVA) to determine the association of phthalate metabolite and isoprostane concentrations with categorical demographic variables, such as maternal country of birth, race, poverty, age, pre-pregnancy BMI and infant sex.

To determine the relationship between different phthalate metabolites with oxidative stress as measured by concentrations of isoprostane, we first calculated Pearson’s correlation coefficients. We fit linear regression models to determine the relationship of prenatal phthalate metabolite concentrations and isoprostane. Isoprostane measured at early or late pregnancy was the dependent variable, while phthalate metabolite concentration measured at the concurrent time point was the independent variable. Separate models were fit for each of the 11 phthalate metabolites and 3 summary measures (∑LMW, ∑HMW and ∑DEHP) during early and late pregnancy. Additionally, for each phthalate metabolite and summary measure, we also created a regression model examining the association of the change in phthalate metabolite concentrations from 13–26 weeks gestation (independent variable) with the change in isoprostane concentrations from 13–26 weeks gestation (dependent variable). Maternal creatinine levels were included as a covariate in these models to adjust for urinary dilution. Years in the U.S., education, parity, pre-pregnancy BMI and child’s sex were included in the models as potential confounders, because they were associated with at least one phthalate metabolite concentration and levels of isoprostane. Time of day of urine collection was also associated with phthalate metabolite concentrations, but we did not include this variable in final models, because it was not associated with isoprostane levels. As a sensitivity analysis, we also performed regression models adjusting for specific gravity instead of creatinine to account for urinary dilution; however, results did not change appreciably, and therefore, we report the results for the “creatinine” models only. All statistical analyses were carried out using R Version 3.2.2 (R Foundation for Statistical Computing, Vienna, Austria, 2013). *p*-values less than 0.05 were considered significant, and *p*-values less than 0.10 were reported as marginally significant.

## 3. Results

### 3.1. CHAMACOS Participants

Demographic characteristics of the CHAMACOS pregnant women are summarized in Table 1. Most of them were Mexican-American from a major agricultural area of Salinas Valley, CA, USA and 50% of them lived less than five years in the U.S. at the time of pregnancy. While relatively young (26.3 years old on average), many were overweight (38.4%) or obese (24.1%). Alcohol use and smoking were not common in this population of women, which was also characterized by low levels of education and income (most within 200% of the poverty level).

### 3.2. Phthalate Exposure

Phthalate exposure as measured by eleven urine metabolites was common. Detection frequencies were above 90% for all phthalate metabolites and reached 99.7% for MEP and 100% for MECPP in CHAMACOS participants. Table 2 shows the distribution of phthalate metabolite and isoprostane concentrations measured in urine during early and late pregnancy. Distributions of phthalate metabolites were similar to those reported in NHANES women of child-bearing age [49]. As expected, MEP had by far the highest concentrations in prenatal urine at both time points among all metabolites.

**Figure 1:**
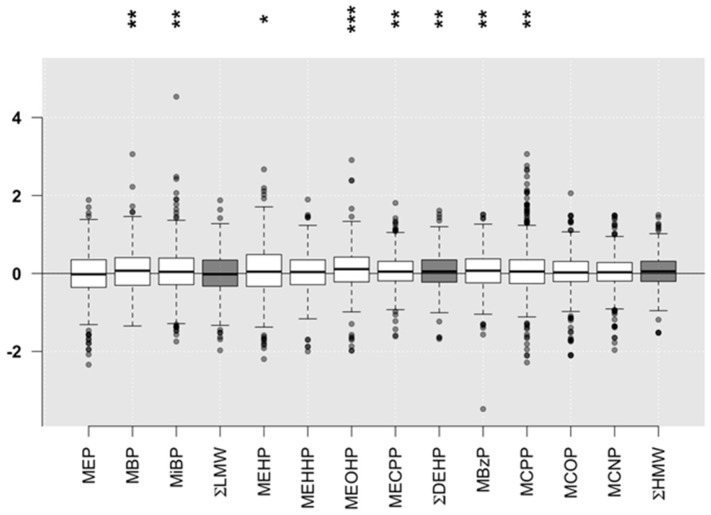
Differences in the phthalate metabolite concentrations (creatinine adjusted) between samples from the same individual collected at 13 and 26 weeks gestation. Differences that are statistically significant are indicated by: * *p* < 0.05, ** *p* < 0.01 and *** *p* < 0.001.

MEP and MBzP concentrations had the highest correlations between early and late pregnancy (*r* = 0.39, *p* = 4.44 × 10^−16^, and *r* = 0.38, *p* = 2.66 × 10^−15^, respectively) among phthalate metabolites. The correlations for other HMW metabolites (MCOP, MCPP, MCNP) were weaker, but still statistically significant (*r* = 0.14–0.21, *p* < 0.05) (Table 2). Statistically-significant, but relatively small differences between early and late pregnancy concentrations were observed for all DEHP metabolites with the exception of MEHHP (Figure 1). Two HMW metabolites were statistically higher later in pregnancy (MBzP and MCPP, *p* < 0.01) in comparison to early pregnancy, but this was not the case for the ∑HMW (*p* > 0.05) (Figure 1).

**Figure 2:**
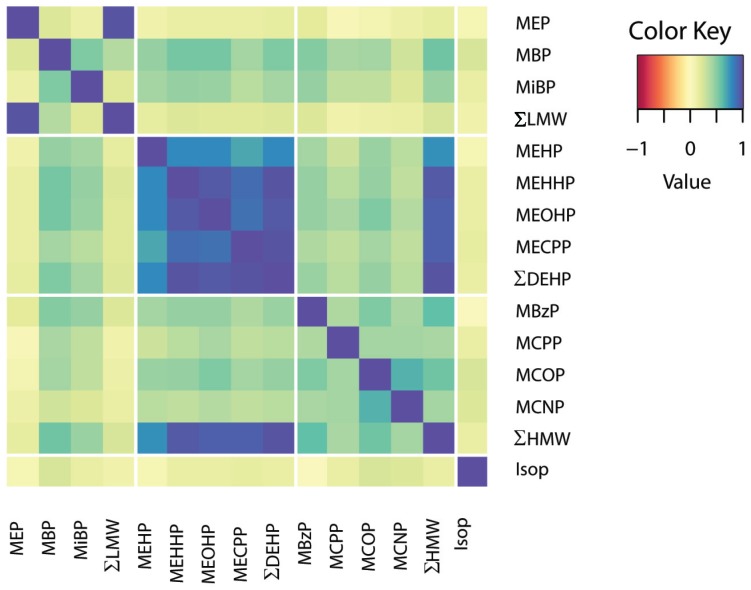
Correlation matrix of concentrations between eleven phthalate metabolites and isoprostane averaged for two time points in early and late pregnancy. Each colored square represents Pearson’s correlation coefficient between different phthalate metabolites. The dark blue squares indicate strong positive correlations with correlation coefficients ranging from 0.5–1.

The heat map in Figure 2 shows the relationship of different creatinine-adjusted phthalate metabolite concentrations with each other. The concentrations of DEHP metabolites were highly correlated with each other. Figure 2 also highlights a strong relationship between ∑HMW and all DEHP metabolite concentrations throughout pregnancy. Among LMW metabolites, ∑LMW was primarily driven by MEP concentrations, while MBP and MiBP were more highly correlated with each other than with MEP.

**Figure 3:**
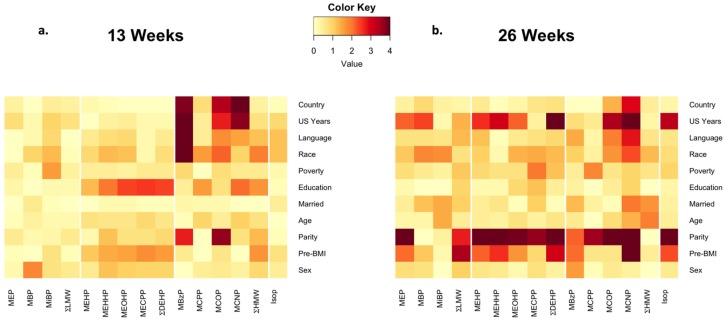
Heatmap of the relationship between phthalate metabolite concentrations and demographic variables at (**a**) 13 and (**b**) 26 weeks gestation. Each colored square represents the −log10 *p*-value for the association of demographic variables (using the same categories as Table 1) with phthalate metabolites concentrations as determined by analysis of variance (ANOVA). The darker squares represent associations with smaller *p*-values, with dark red representing the most significant associations (*p* ~ 1 × 10^−4^). Each model is adjusted for creatinine by including it as a covariate.

Figure 3 shows the heat map illustrating the relationship between phthalate metabolite concentrations and demographic variables. Effects of maternal age, sex of the child, marriage and poverty status were negligible at 13 weeks of pregnancy. However, determinants of acculturation, such as years in the U.S., country of birth, race and primary language, became surprisingly strong for MBzP. This relationship was still noticeable, but weaker at 26 weeks. DEHP metabolites appear moderately affected by maternal level of education and pre-pregnancy BMI, but this was limited to 13 weeks. In contrast, at 26 weeks, the strongest relationship with parity was seen for both DEHP and HMW metabolites. Detailed parameters for these relationships can be found in Table S1 and Table S2. There was no meaningful association between most of these demographic variables with the LMW metabolite concentrations at early pregnancy. However, MEP and ΣLMW concentrations appear to be related to parity, pre-pregnancy BMI and years in the United States during late pregnancy.

### 3.3. Isoprostane

The distribution of isoprostane concentrations in urine samples shows an almost 20% increase at 26 weeks in comparison to 13 weeks (the median 3.6 *vs.* 4.6 µg/g creatinine, respectively; Table 1). However, the ranges were similar, from 0.3–29.3 µg/g creatinine at early pregnancy and from 0.4–25.0 µg/g creatinine later in the pregnancy. The correlation of isoprostane concentrations between the two time points was modest (*r* = 0.18, *p* = 0.03). No significant associations between demographic variables and isoprostane concentrations were observed at early pregnancy (Figure 3). At late pregnancy, isoprostane levels were significantly associated with years in the United States, parity and pre-pregnancy BMI.

### 3.4. Phthalates and Isoprostane

Results of the regression analysis examining the associations of phthalate metabolite concentrations with isoprostane concentrations at 13 and 26 weeks gestation are shown in Table 3. Effect estimates in crude models were similar to those in the final adjusted models reported here. Most of the relationships at 13 weeks were not significant with the exception of two HMW metabolites; MCOP and MCNP were significantly positively associated with isoprostane with a 0.178 µg/g creatinine (standard error (*SE*) = 0.059) and a 0.152 µg/g creatinine (*SE* = 0.063) increase, respectively, per 10-fold increase in metabolite level. However, the picture was quite different at 26 weeks of pregnancy. Specifically, many of the LMW and HMW metabolite concentrations were significantly associated with isoprostane concentrations. For instance, while the relationship with MEP was moderate (*p* = 0.04), the relationship of isoprostane concentrations with other LMW metabolite concentrations, as well as ∑LMW were stronger (*p*-values between 0.0004 and 0.01). Using a linear model that accounts for creatinine as a covariate, as well as other demographic covariates, the correlation coefficient relating MBP and isoprostane concentrations at the 26-week visit was modest, *r* = 0.11, but significant (*p* = 0.009). DEHP metabolites were not associated with isoprostane in the cross-sectional models at 13 and 26 weeks (Table 3).

Among non-DEHP HMW phthalate metabolites, only MBzP was not significantly associated with isoprostane concentrations at either time point. The relationship between MCPP with isoprostane was strong at 26 weeks (β(*SE*): 0.125(0.046); *p* = 0.007), but not at 13 weeks (β(*SE*): 0.33(0.040); *p* = 0.41). The opposite was the case for MCOP concentrations (*p* = 0.003 at 13 weeks and *p* = 0.11 at 26 weeks). In contrast, MCNP and isoprostane concentrations were significantly associated at both early and late pregnancy (*p* = 0.02 at both time points).

When we modeled the relationship of the change in phthalate metabolite concentrations with the change in isoprostane levels from early to late pregnancy (Table 3), we found a positive association for MEP (*p* = 0.03). The results for ∑LMW metabolites (*p* = 0.01) appear to be driven by MEP, because MBP was only borderline significant (*p* = 0.068), and MiBP was not significant. For DEHP metabolites, there was a suggestive trend of increasing isoprostane levels with higher metabolite concentrations, but the associations did not reach statistical significance when metabolites were considered individually. However, the relationship between changes over pregnancy in isoprostane and ∑DEHP metabolites (*p* = 0.046) were modest, but statistically significant. We saw a similar trend for HMW metabolites where there were suggestive trends of a positive relationship with isoprostanes for individual metabolites, but the association only reached statistical significance for the sum of metabolites (∑HMW; *p* = 0.049).

The difference between results with the individual time point models (13 and 26 week) *versus* the change models can be explained as follows: the single time point models show correlation between phthalate metabolites and isoprostane concentrations at a given time (static), whereas the change model includes two time points and reflects correlations of the change in the two measures over time (dynamic). Demonstrating that two variables change over time in the same way provides additional evidence of a common association. The change model also incorporates a larger number of measurements, providing increased power to detect associations compared to the static single time point model.

Importantly, beta coefficients were consistently positive, indicating that isoprostane levels in CHAMACOS pregnant women tended to increase with higher phthalate metabolite concentrations.

## 4. Discussion

In this study, we examined the association of prenatal phthalate exposure during early and late pregnancy in CHAMACOS women with 8-isoprostane, a biomarker of lipid peroxidation resulting from oxidative stress. We observed a broad range of urinary phthalate metabolite concentrations in this Mexican-American rural cohort. The relationship of isoprostane and phthalate metabolite concentrations was not consistent over pregnancy. While it was significant for only two HMW metabolites (MCOP and MCNP) at 13 weeks of pregnancy, all three LMW metabolites (MEP, MBP and MiBP) were associated with oxidative stress later in the pregnancy when the levels of isoprostanes were 20% higher. 

Our findings of high detection and a broad distribution of phthalate metabolite concentrations are consistent with previous reports for NHANES [32,39,42,50] and several other studies [26,36,49,51], as well as the California Environmental Biomonitoring Program [52]. Patterns of temporal variability for phthalate metabolite concentrations over pregnancy, including higher temporal variability in DEHP metabolites and more moderate temporal variability for MEP, were similar to those reported in other studies, as well [36,53,54]. MEP, reflective of the use of DEP in perfumes, deodorants, shampoo and soaps [55,56], was the most common metabolite in CHAMACOS pregnant women. DBP and di-isobutyl phthalate are also used in personal care products and cosmetics. These three LMW phthalates have been associated with a number of health conditions related to endocrine disruption, such as obesity, asthma, preterm birth and neurobehavioral problems [36,57,58]. MEP was also common in a cohort of men and women from Boston [51]. However, in that cohort, it was closely followed in concentrations by MBP, while in CHAMACOS women, the difference was an order of magnitude. In the Boston study, cologne users had MEP urine concentrations 167% higher than non-users. The difference was 28% for lotion users and also noted for women who used nail polish in the last 24 h before urine collection. Overall, women who reported using more personal care products had higher urinary concentrations of the two measured phthalate metabolites, MEP and MBP, and of three parabens [51]. In another study of adolescent girls (Health and Environmental Research in Make-up Of Salinas Adolescents; HERMOSA) from the same area of Salinas Valley, CA, where our CHAMACOS is located, urine measurements after a three-day replacement of their usual personal care products with those with lower levels of phthalates resulted in a significant decrease of urine concentrations by 27% for MEP, but no change in MBP and MiBP [59], possibly because these latter chemicals are in other products [60,61,62,63]. 

In our cohort, MEHP, a monoester metabolite of DEHP, was present in lower concentrations than reported in other studies [36,50] in the majority of pregnant CHAMACOS women. It was proposed that the increased ratio of urine concentrations of oxidized DEHP metabolites (MEHHP, MEOHP and MECPP) to MEHP may be evidence of inter-individual differences in efficiency of conversion of MEHP to less toxic metabolites that can be readily excreted [64,65,66]. In primarily Mexican-American CHAMACOS cohort, we observed a significant increase in concentrations of HMW metabolites, especially MBzP, in association with parameters of acculturation, such as race, primary language spoken at home (English *vs.* Spanish), country of birth (USA *vs.* Mexico) and more years living in the U.S. Some differences in the concentrations of phthalate metabolites related to ethnic background, socioeconomic status (SES) and predominant types of exposure were also noted in several other cohorts from the U.S., Puerto-Rico, Taiwan and Korea [4,36,58,67]. Another factor that could possibly contribute to the difference is the diet. CHAMACOS women and their families commonly consumed a number of typical Mexican foods and drinks, such as chalupas or flautas (type of tacos), quesadillas, plantains, *etc.*, but in general their diet was Americanized, containing frequent consumption of soft drinks and fast food, especially for those who were in the country the longest [68]. In the future, it will be interesting to explore this relationship more thoroughly by examining the associations of phthalates with other measures of acculturation and diet.

In our study, we observed a substantial increase in 8-isoprostane concentrations at 26 weeks of pregnancy in comparison with earlier pregnancy. This finding is consistent with studies that have demonstrated a gradual increase of systemic oxidative stress as pregnancy progresses [36]. The level of oxidative stress is an important factor in embryogenesis, as well as for pregnancy and normal birth. Pregnancy itself is a state of higher oxidative stress levels; and 8-isoprostane may be a useful marker for the risk for pregnancy complications [69]. It has been reported that isoprostane levels were significantly increased in pregnant women in relation to healthy non-pregnant women and were higher during the second and third trimester of pregnancy [69,70]. Isoprostane levels in CHAMACOS pregnant women appear to be higher than reported by similar methods in pregnant women from Europe [71]. This may be related to a high prevalence of obesity in this cohort that was a significant factor associated with isoprostane at late pregnancy, as well as other factors, such as diet or acculturation. However, it is more difficult to make a comparison with some other cohorts that either used alternative methods of isoprostane measurements [72] or do not report similar adjustments. However, it does appear that at least some of the CHAMACOS pregnant women had noticeably higher levels of isoprostane, especially in late pregnancy, than in the Boston and Puerto Rico cohorts [33,36]. Moreover, complications of pregnancy, such as preeclampsia, have been associated with elevated oxidative stress in comparison to normal pregnancy cross-sectionally [71].

Several studies have reported that oxidative stress during pregnancy is predictive of adverse outcomes. For instance, in a study of 503 healthy pregnant women with samples collected at 24–26 weeks of gestation and prospectively followed through postpartum [73], women with significantly higher plasma 8-isoprostane levels were at higher risk of developing preeclampsia and delivering small-for-gestational age infants. Another study indicated elevated levels of prostaglandins, such as 8-isoprostane, to be associated with an increased risk of preterm birth [74]. These findings demonstrate that increased maternal oxidative stress is associated with subsequent pregnancy complications [73]. The vasoconstrictive and inflammatory properties of oxidative stress may cause maternal endothelial dysfunction and leukocyte activation, which may elucidate the pathogenesis of these pregnancy complications. 

Previously, positive relationships were found between biomarkers of oxidative stress, including 8-isoprostane, malondialdehyde (MDA) and 8-oxo-2′-deoxyguanosine (8-oxo-dG), with phthalate metabolite concentrations [4,26,32,75]. In our cohort, at 13 weeks of pregnancy, only two HMW metabolites (MCOP and MCNP) were significantly associated with 8-isoprostane. This relationship persisted for MCNP at 26 weeks when MCPP, another HMW metabolite, was also associated with 8-isoprostane. However, the relationship with MCOP was no longer significant later in pregnancy. The most striking change between early and late pregnancy in CHAMACOS women in regards to the 8-isoprostane was observed for LMW metabolites, showing consistently strong associations for MEP, MBP and MiBP at 26 weeks, but not at 13 weeks. However, the same was not seen for MBzP metabolites. As in our study, Ferguson and colleagues [4] report a highly significant positive relationship between phthalate metabolites with 8-isoprostane in pregnant women. However, in that study, relationships for all phthalate metabolites were significant, possibly because of a larger number of isoprostane measurements providing more statistical power. It may be also explained by ethnic and SES differences between cohorts or perhaps dietary differences in exposure to DEHP in the urban Boston cohort compared to CHAMACOS [76,77].

This study has several strengths and some limitations. It was performed in a well-characterized, large minority cohort of pregnant women from Salinas Valley, CA, a major agricultural area in the United States. Given that CHAMACOS cohort is relatively homogeneous with regards to race and social class, it potentially reduces the impact of unaccounted confounding. Measurements of a validated biomarker of oxidative stress, urinary isoprostane, were performed twice during pregnancy allowing for comparison of the relationship with phthalate metabolites in early and late pregnancy in the same participants. As for most biomarker studies, we cannot completely eliminate the possibility of potential residual confounding, misclassification of exposure and outcome variables and selection bias, despite our best efforts to address them. One potential challenge is that phthalates may not be the only chemical affecting oxidative stress in the CHAMACOS cohort. However, we have already characterized exposures to PBDEs and BPA (among others) and did not see significant associations with BPA, another endocrine disruptor with similar patterns of exposure to phthalates. In the future, it would be desirable to apply an exposome-type approach to characterize the combined effects of many exposures simultaneously. To this effect, we recently initiated a metabonomic study of samples from the CHAMACOS pregnant women. Finally, given that participants in our study were Mexican-Americans from a low SES rural cohort, the interpretation of the findings may not be completely applicable to other ethnic groups or urban populations with a higher income and different life style.

## 5. Conclusions

In conclusion, average phthalate metabolite concentrations in Mexican-American pregnant women from a rural area remain fairly constant over pregnancy. The relationship of the biomarker of oxidative stress 8-isoprostane with low molecular weight phthalate metabolites was significant in late, but not early pregnancy. We also observed a statistically-significant, but not consistent association of 8-isoprostane with HMW metabolites at both early and late pregnancy, while DEHP metabolites were marginally associated with oxidative stress only if the sum of these metabolites was considered in the models. The oxidative stress mechanism related to phthalate exposure during pregnancy is especially important, as it is one of the critical pathways that may lead to adverse health outcomes, such as preterm delivery. These new data for a large rural minority cohort add to existing information about molecular mechanisms of phthalate exposure in different populations.

## Figures and Tables

**Table 1:** Demographic characteristics of Center for Health Assessment of Mothers and Children of Salinas (CHAMACOS) mothers (1999–2000).

Characteristic	Mothers with Phthalate Data * (*N* = 433) *N* (%)	Mothers with Isoprostane Data* (*N* = 196) *N* (%)
Pre-pregnancy Weight Status	-	-
Normal	156 (36.8)	59 (30.1)
Underweight	3 (0.7)	1 (0.5)
Overweight	163 (38.4)	84 (42.9)
Obese	102 (24.1)	52 (26.5)
Age at Delivery	-	-
18–24	189 (43.8)	70 (35.7)
25–29	136 (31.5)	73 (37.2)
30–34	70 (16.2)	32 (16.3)
35–45	37 (8.6)	21 (10.7)
Education	-	-
≤6th grade	186 (43)	89 (45.4)
7–12th grade	154 (35.6)	71 (36.2)
≥High School Graduate	93 (21.5)	36 (18.4)
Years in U.S.	-	-
≤1	107 (24.7)	45 (23)
2–5	111 (25.6)	48 (24.5)
6–10	98 (22.6)	60 (30.6)
11+	66 (15.2)	30 (15.3)
Entire life	51 (11.8)	13 (6.6)
Poverty Status	-	-
At or below poverty	270 (62.4)	132 (67.3)
Poverty-200%	148 (34.2)	57 (29.1)
>200% poverty	15 (3.5)	7 (3.6)
Alcohol Use during Pregnancy	-	-
No	403 (94.6)	183 (94.8)
Yes	23 (5.4)	10 (5.2)
Smoking during Pregnancy	-	-
No	410 (94.7)	188 (95.9)
Yes	23 (5.3)	8 (4.1)
Parity	-	-
0	144 (33.3)	59 (30.1)
≥1	289 (66.7)	137 (69.9)

* Total number of observations vary due to missing data.

**Table 2:** Distribution of phthalate metabolite concentrations at 13 and 26 weeks gestation.

Exposure *	13 Weeks (*N* = 432)				26 Weeks (*N* = 417)				Correlation	*p*-Value
	Median	IQR	5th	95th	Median	IQR	5th	95th		
MEP	161.8	(67.3, 435.1)	24.3	1618.6	153.3	(65.6, 376.3)	22.2	1262.1	0.389	4.44 × 10^−16^
MBP	18.4	(9.1, 37.9)	3.7	102.4	22.6	(11.7, 42.6)	5.0	121.6	0.211	1.76 × 10^−5^
MiBP	2.4	(1.1, 4.5)	0.2	14.0	2.8	(1.4, 5.2)	0.4	14.4	0.267	4.46 × 10^−8^
ΣLMW	211.9	(100.7, 524.4)	40.0	1806.8	217.8	(113.7, 451.4)	43.2	1506.3	0.371	9.77 × 10^−15^
MEHP	3.0	(1.4, 6.4)	0.2	17.7	3.6	(1.9, 6.7)	0.4	18.0	0.199	5.04 × 10^−5^
MEHHP	12.6	(6.8, 24.7)	2.5	76.9	15.7	(8.3, 28.1)	3.3	66.2	0.233	2.02 × 10^−6^
MEOHP	8.7	(4.7, 16.7)	1.6	47.2	12.1	(6.8, 21.1)	2.8	48.4	0.204	3.37 × 10^−5^
MECPP	22.0	(13.7, 39.7)	5.9	104.0	25.8	(16, 45.6)	8.5	97.5	0.241	8.79 × 10^−7^
ΣDEHP	46.0	(27.6, 84.3)	11.8	242.3	57.8	(33.2, 99.3)	16.1	235.5	0.231	2.35 × 10^−6^
MBzP	6.6	(3.1, 12.7)	0.9	32.1	7.6	(4.3, 14)	1.5	38.1	0.378	2.66 × 10^−15^
MCPP	1.8	(1, 2.9)	0.1	6.4	2.1	(1.2, 3.2)	0.2	6.4	0.154	1.76 × 10^−3^
MCOP	2.8	(1.7, 4.6)	0.5	10.7	3.2	(2.1, 5)	0.8	9.6	0.138	5.30 × 10^−3^
MCNP	1.8	(1, 2.7)	0.4	7.2	1.9	(1.3, 2.8)	0.6	5.8	0.206	2.80 × 10^−5^
ΣHMW	65.7	(37.8, 110.4)	17.6	303.8	79.5	(46.8, 126.4)	24.2	270.5	0.244	6.37 × 10^−7^
Isoprostane **	3.6	(2.2, 5.0)	1.0	10.8	4.6	(3.1, 6)	1.2	8.9	0.176	3.34 × 10^−2^

* All units are µg/g creatinine; ** *N* = 166 for 13 weeks and *N* = 180 for 26 weeks.

**Table 3:** Regression models of phthalate metabolite concentrations with isoprostane levels at 13 and 26 weeks gestation ^a^.

Phthalate Metabolite (µg/g Creatinine)	13 Weeks (*n* = 166)			26 Weeks (*n* = 180)			Change (*n* = 150)		
	β	95% CI	*p*-Value	β	95% CI	*p*-Value	β	95% CI	*p*-Value
MEP	0.045	(−0.031, 0.121)	0.2486	0.074	(0.003, 0.145)	0.0417	0.110	(0.014, 0.206)	0.0271
MBP	0.064	(−0.028, 0.156)	0.1721	0.183	(0.083, 0.283)	0.0004	0.094	(−0.006, 0.194)	0.0684
MiBP	−0.002	(−0.088, 0.084)	0.9665	0.097	(0.023, 0.171)	0.0108	0.059	(-0.031, 0.149)	0.2012
ΣLMW	0.056	(−0.028, 0.140)	0.2007	0.109	(0.029, 0.189)	0.0087	0.144	(0.036, 0.252)	0.0099
MEHP	0.006	(−0.080, 0.092)	0.8949	0.057	(−0.023, 0.137)	0.1643	0.073	(−0.019, 0.165)	0.1239
MEHHP	0.066	(−0.048, 0.180)	0.2526	0.068	(−0.018, 0.154)	0.1271	0.101	(-0.009, 0.211)	0.0768
MEOHP	0.064	(−0.036, 0.164)	0.2160	0.072	(−0.018, 0.162)	0.1220	0.092	(-0.014, 0.198)	0.0910
MECPP	0.087	(−0.050, 0.224)	0.2151	0.074	(−0.040, 0.188)	0.2002	0.130	(−0.005, 0.265)	0.0613
ΣDEHP	0.075	(−0.052, 0.202)	0.2499	0.080	(−0.026, 0.186)	0.1349	0.131	(0.004, 0.258)	0.0464
MBzP	0.045	(−0.059, 0.149)	0.3983	0.012	(−0.074, 0.098)	0.7805	0.076	(−0.032, 0.184)	0.1710
MCPP	0.033	(−0.045, 0.111)	0.4142	0.125	(0.035, 0.215)	0.0072	0.003	(−0.052, 0.112)	0.4728
MCOP	0.178	(0.062, 0.294)	0.0031	0.090	(−0.020, 0.200)	0.1119	0.078	(−0.045, 0.201)	0.2188
MCNP	0.152	(0.029, 0.275)	0.0166	0.136	(0.021, 0.250)	0.0211	0.110	(−0.014, 0.233)	0.0832
ΣHMW	0.088	(−0.050, 0.226)	0.2119	0.092	(−0.026, 0.209)	0.1300	0.142	(0.002, 0.283)	0.0487

^a^ Covariates in each regression model included creatinine and categorical variables for years in U.S., education level, parity, pre−pregnancy BMI and sex of child.

## Supplementary Materials

The following are available online at www.mdpi.com/2305-6304/4/1/7/s1. Table S1: Mean phthalate metabolite concentrations (µg/L) during early pregnancy by maternal demographic variables. Table S2: Mean phthalate metabolite and isoprostane concentrations (µg/L) during late pregnancy by maternal demographic variables.

## Abbreviations

The following abbreviations are used in this manuscript:

BMI: body mass index
CDC: Centers for Disease Control and Prevention
CHAMACOS: Center for Health Assessment of Mothers and Children of Salinas
CHAP: Chronic Hazard Advisory Panel
DBP: dibutyl phthalate
DEHP: di-2-ethylhexyl phthalate
DEP: diethyl phthalate
HMW: high molecular weight
LMW: low molecular weight
LOD: limit of detection
MBP: mono-*n*-butyl phthalate
MBzP: monobenzyl phthalate
MCNP: monocarboxynonyl phthalate
MCOP: monocarboxyoctyl phthalate
MCPP: mono(3-carboxypropyl) phthalate
MDA: malondialdehyde
MECPP: mono(2-ethyl-5-carboxypentyl) phthalate
MEHP: mono(2-ethylhexyl) phthalate
MEHHP: mono(2-ethyl-5-hydroxyhexyl) phthalate
MEOHP: mono(2-ethyl-5-oxohexyl) phthalate
MEP: monoethyl phthalate
MiBP: mono-isobutyl phthalate
NHANES: National Health and Nutrition Examination Survey
SE: standard error
SES: socioeconomic status
QA/QC: quality assurance/quality control
8-oxo-dG: 8-Oxo-2′-deoxyguanosine
