# Supplementary material for: Urinary Phthalate Metabolites and Biomarkers of Oxidative Stress in a Mexican-American Cohort: Variability in Early and Late Pregnancy
Source: Toxics. 2016 Mar 14;4(1):7. doi: 10.3390/toxics4010007 (PMC5171220; doi:10.3390/toxics4010007)
Supplement: Supplementary file 1 [file toxics-04-00007-s001.pdf]

Supplementary Materials: Urinary Phthalate Metabolites and Biomarkers of Oxidative Stress in a Mexican-American Cohort: Variability in Early and Late Pregnancy

Nina Holland *, Karen Huen, Vy Tran, Kelly Street, Brian Nguyen, Asa Bradman and Brenda Eskenazi

Table S1. Mean phthalate metabolite concentrations (μg/L) during early pregnancy by maternal demographic variables.

Characteristic	Frequency	MBzP	∑HMW
Country of birth			
US	57	17.23	88.75
Mexico	363	9.64	110.13
Other	8	67.06	232.18
Years in US			
≤1	105	9.71	118.00
2–5	110	8.65	93.91
6–10	98	10.23	116.67
11+	66	16.63	122.33
Entire life	49	19.32	95.22
Language spoken at home			
Spanish	379	9.89	109.23
Both	20	10.74	70.61
English	24	41.94	142.51
Other	5	9.86	132.58
Race			
White	7	13.11	73.99
Latino	415	10.40	107.77
Other	6	30.16	275.23

Table S2. Mean phthalate metabolite and isoprostane concentrations (μg/L) during late pregnancy by maternal demographic variables.

Characteristic	Frequency *	∑LMW	∑HMW	∑DEHP	Frequency **	Isoprostanes
Parity						
0	136	518.76	88.83	69.53	52	5.20
≥1	273	427.08	129.47	104.11	125	5.01
Pre-pregnancy Weight Status						
Normal	3	211.44	72.28	60.40	1	1.17
Underweight	148	377.95	112.95	91.80	54	4.92
Overweight	152	502.07	105.95	82.30	75	5.68
Obese	97	531.17	140.19	113.76	47	4.35
Years in US						
≤1	103	550.67	100.32	79.33	40	6.08
2–5	104	420.44	98.18	77.97	43	4.20
6–10	94	524.29	116.07	95.11	56	5.58
11+	64	390.57	171.39	143.88	25	3.73
Entire life	44	282.27	113.69	78.35	13	5.20

* Frequency for women with phthalate metabolite measurements; ** Frequency for women with isoprostane measurements.
